# Patchiness of phytoplankton and primary production in Liaodong Bay, China

**DOI:** 10.1371/journal.pone.0173067

**Published:** 2017-02-24

**Authors:** Shaofeng Pei, Edward A. Laws, Haibo Zhang, Siyuan Ye, Hongming Yuan, Haiyue Liu

**Affiliations:** 1 Key Laboratory of Coastal Wetland Biogeosciences, China Geological Survey, Qingdao, China; 2 Function Laboratory for Marine Geology, National Oceanography Laboratory, Qingdao, China; 3 Department of Environmental Sciences, School of the Coast and Environment, Louisiana State University, Baton Rouge, Los Angeles, United States of America; 4 College of Chemistry and Chemical Engineering, Ocean University of China, Qingdao, China; University of Connecticut, UNITED STATES

## Abstract

A comprehensive study of water quality, phytoplankton biomass, and photosynthetic rates in Liaodong Bay, China, during June and July of 2013 revealed two large patches of high biomass and production with dimensions on the order of 10 km. Nutrient concentrations were above growth-rate-saturating concentrations throughout the bay, with the possible exception of phosphate at some stations. The presence of the patches therefore appeared to reflect the distribution of water temperature and variation of light penetration restricted by water turbidity. There was no patch of high phytoplankton biomass or production in a third, linear patch of water with characteristics suitable for rapid phytoplankton growth; the absence of a bloom in that patch likely reflected the fact that the width of the patch was less than the critical size required to overcome losses of phytoplankton to turbulent diffusion. The bottom waters of virtually all of the eastern half of the bay were below the depth of the mixed layer, and the lowest bottom water oxygen concentrations, 3–5 mg L^–1^, were found in that part of the bay. The water column in much of the remainder of the bay was within the mixed layer, and oxygen concentrations in both surface and bottom waters exceeded 5 mg L^–1^.

## Introduction

The temporal and spatial variability of plankton populations in the ocean has been a topic of interest to oceanographers for more than a century [1]. It has been argued that the patchiness of plankton distributions is critical to the survival of larval fish [2–4], and the patchiness of environmental conditions in both freshwater and marine systems is typically cited as the principal explanation for Hutchinson’s [5] paradox of the plankton [6, 7], i.e., the diversity of species that coexist in the same habitat. The patches of interest range in size from mesoscale eddies with dimensions of tens of kilometers [8, 9] to excreted nutrient plumes with dimensions on the order of 10–100 μm in the wake of zooplankton [10, 11]. Empirical and theoretical studies have focused on the mechanisms that produce patches and the mechanisms that destroy them [12–15].

Among the principal mechanisms that create patches of phytoplankton are water column stratification and spatial variability in the supply of nutrients essential for phytoplankton growth [16, 17]. Estuaries are naturally characterized by horizontal and vertical gradients in salinity, and during the summer many estuaries are thermally stratified. There is a natural vertical gradient in the flux of photosynthetically active radiation (400–700 nm, PAR) due to the scattering and absorption of light by particles and dissolved substances in the water column. Furthermore, the input of allochthonous nutrients via stream discharges creates a natural gradient in the supply of nutrients from the head to the mouth of the estuary [18, 19]. The combination of vertical gradients in PAR and water density and horizontal gradients in nutrient supply can lead to high rates of oxygen production and consumption in surface waters and bottom waters, respectively. The development of hypoxia and anoxia associated with these conditions has become a serious problem in many estuarine systems impacted by anthropogenic nutrient loading [20, 21].

**Fig 1:**
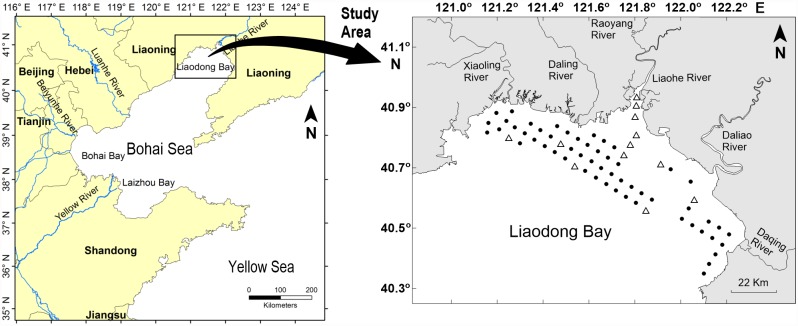
Study area, sampling stations (● and △) and ^14^C incubation stations (△) in Liaodong Bay, China.

Liaodong Bay, one of the three major bays in the Bohai Sea, is located in the most northern sector of the Bohai Sea near the Liaodong Penisula in northeastern China (Fig 1). The cross section between Liaodong Bay and the Bohai Sea is approximately 160 km wide. The tidal range is 2.7 m at the head and 0.8 m at the mouth of the bay (p. 164 in [22]). The bay receives the discharge of several large rivers, including the Shuangtaizi River (now known as the Liaohe River), Xiaoling River, Daling River, Daliao River, Luan River, Liugu River, and Fuzhou River, as well as some smaller streams [23]. In recent years rapid economic development has been associated with large discharges of industrial and domestic wastewater to the bay that have created eutrophication problems, including algal blooms and red tides [24, 25].

The degree of eutrophication in the bay has been mapped based on the status of the benthic macroinvertebrate community [24] and with use of a eutrophication index that is the product of the concentrations of dissolved inorganic nitrogen, phosphate, and chemical oxygen demand [24, 26]. However, there have been surprisingly few studies of photosynthetic rates or phytoplankton biomass in Liaodong Bay and their relationships to environmental conditions, in contrast to comparatively well-studied Chinese bays such as Laizhou Bay [27] and Jiaozhou Bay [28, 29]. The goal of the present study was to map the distribution in Liaodong Bay of the factors that limit phytoplankton production—nutrient concentrations, PAR, and temperature—and to relate the vertical and horizontal variability of these factors to the distribution of photosynthetic rates and phytoplankton biomass. An understanding of the patterns of photosynthesis in the bay and the stability of the water column as a function of temperature and salinity made it possible to explain much of the variability of oxygen concentrations in Liaodong Bay and the susceptibility of the bay to hypoxia. In addition, because many harmful algal species have been observed within phytoplankton patches and thin layers [30–32], investigation of the mechanisms of formation and environmental drivers of these patches may enhance understanding of the frequency of harmful algal blooms (HABs) both locally and globally.

In this study, we tested the hypothesis that the distribution of phytoplankton in the bay was independent of macronutrient concentrations and instead was determined by a combination of temperature, irradiance, and physical processes. More specifically, we hypothesized that patches of high phytoplankton biomass were able to develop in areas where the temperature and irradiance facilitated rapid growth of phytoplankton and where physical mixing was not so vigorous as to preclude patch formation.

Given the increasing rate of allochthonous nutrient loading of coastal ecosystems from land runoff, it seems probable that bottom-up controls on the size and frequency of phytoplankton patches in coastal waters will continue to shift from nutrients to light and temperature, with potentially profound implications for fish production [2–4], the occurrence of harmful algal blooms (HABs) [33–35], and hypoxia [36]. Lessons learned from studies of estuaries such as Liaodong Bay may therefore be broadly relevant to a variety of issues in coastal ecosystems in the future.

## Materials and methods

### Study area and sampling stations

The study area and 66 sampling stations in Liaodong Bay are shown in Fig 1. Because the water depth was less than 5 m at most of the stations, water samples were collected with Nansen bottles from the surface and bottom at each station by a Chinese Fishery research vessel on three cruises authorized by Qingdao Institute of Marine Geology, China Geological Survey during June and July of 2013. Specific permission was not required because Chinese government allows research investigation in the whole Bohai Sea, including our study area—Liaodong Bay. Our field studies did not involve any endangered or protected species. Most stations were sampled once, but seven randomly selected stations were sampled on two occasions to provide a measure of temporal variability. The water was then filtered through pre-combusted (450°C for 6 h) Whatman GF/F filters (25-mm diameter). The filtrates were preserved with 0.3% chloroform in polyethylene bottles pretreated with 10% HCl for 24 h and then stored in an icebox at –25°C prior to laboratory nutrient analyses. The GF/F filters were stored in the icebox of a refrigerator for later analysis of Chl *a*.

Temperature (T) was measured *in situ* with a portable dissolved oxygen meter (YSI NC41-Pro 20, Xylem Inc. USA). Salinity (S) and Secchi depths were measured with a digital salinometer (SA287, Aipu Instrument & Equipment Co, LTD, China) and Secchi disk [37], respectively. Alkalinity was determined following the procedures and using the equations in Strickland and Parsons [38], and pH was measured with a pH meter (S470 SevenExcellence^™^, Mettler-Toledo, LLC, USA) with a resolution of ± 0.002. Dissolved inorganic carbon (DIC) concentrations were calculated from the values of total alkalinity and salinity using the equations in Strickland and Parsons [38]. Concentrations of suspended particulate matter (SPM) were determined from the weight of material collected on a filter [39]: water samples were filtered through pre-dried and weighed cellulose acetate filters (Ø 47 mm, 0.45 μm), and then the filters were dried and weighed again to determine the change in weight.

### In situ ^14^C incubations for measuring primary productivity

Primary production measurements were carried out at six randomly selected stations in Liaodong Bay on 13 July 2013 (Fig 1) and at six stations along the Liaohe River estuary on 30 July 2013 using the ^14^C tracer method as described by Pei et al. [40]. Photosynthetic assimilation numbers (i.e., light-saturated ratios of photosynthesis to Chl *a*, *PB M*) were calculated based on the estimated photosynthetic rates and concentration of Chl *a* at 12 incubation stations. The previous study of Pei et al. [40] showed that photosynthetic rates normalized to Chl *a* concentrations were positively correlated with temperature at temperatures between 22.5°C and roughly 26–27°C but declined precipitously at higher temperatures. A third-order polynomial function of temperature gave a satisfactory fit to the data (*R*^2^ = 0.74). Using the third-order polynomial function of temperature fit to the *PB M* values at 12 stations, Pei et al. [40] estimated *PB M* values and areal photosynthetic rates at all 66 stations.

### Other physical and chemical measurements

Chemical analyses were carried out using the methodologies of Shen et al. [41] and Pei et al. [42]: the indophenol blue method for ammonia (NH_4_-N), cadmium-copper reduction method for nitrate (NO_3_-N) plus nitrite (NO_2_-N), the Griess-Ilosvay reaction for NO_2_-N, the phospho-molybdate complex method for phosphate (PO_4_-P), and the silico-molybdate complex method for silicate (SiO_3_-Si). Dissolved inorganic nitrogen (DIN) was calculated as the sum of NO_3_-N, NO_2_-N, and NH_4_-N. To avoid polymerization of silicon, samples were thawed for at least three hours prior to analysis [43]. All nutrient analyses were carried out using a continuous flow AutoAnalyzer (QuAAtro, Bran+Luebbe GmbH Co.). Chlorophyll *a* was extracted from GF/F filter samples overnight in methanol and then measured with a spectrophotometer (Model TU-1901) using the equations of Jeffrey and Humphrey [44].

## Results

The mean and median depths of the bay at the 66 stations we sampled were 4.8 and 5.1 m, respectively, and the range of depths was 0.5–9.4 m. The surface and bottom salinities averaged 22 and 25, respectively. The corresponding mean temperatures were 24.9 and 23.8°C, respectively. Measurements of nutrient, Chl *a* concentrations, and environmental parameters on two different days at the seven randomly selected stations were compared with paired *t*-tests. The differences were not significant at *p* = 0.05. We concluded that temporal variability during the timeframe of this study was small and therefore combined the results from all three cruises in our analysis of the data.

### Patchiness of surface waters

**Fig 2:**
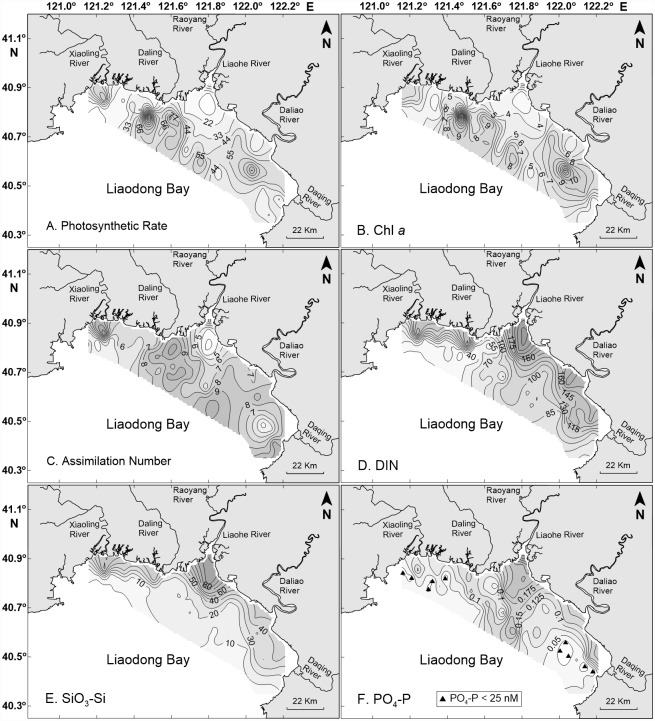
Contour maps of photosynthetic rates (Panel A, in mg C m^–3^ h^–1^), concentrations of Chl *a* (Panel B, in mg m^–3^) and assimilation numbers (Panel C, in mg C mg^–1^ Chl *a* h^–1^), dissolved inorganic nitrogen (DIN, Panel D, in μM), silicate (SiO_3_-Si, Panel E, in μM), and phosphate (PO_4_-P, Panel F, in μM). Triangles in F denote stations with phosphate concentrations less than 25 nM.

Contour maps of areal photosynthetic rates (Fig 2A) and concentrations of Chl *a* (Fig 2B) revealed the presence of several patches of high biomass and production with horizontal dimensions of roughly 10 km. There was a minor peak of Chl *a* concentrations and areal photosynthetic rates off the mouth of the Xiaoling River. The major areas of high production and biomass consisted of two partially merged patches roughly 20 km from the mouth of the Daling River, one directly offshore and the other to the southeast, and of two other partially merged patches, one about 40 km to the southeast of the mouth of the Liaohe River and the other in a region roughly 25 km equidistant from the mouths of the Daliao and Daqing rivers. Consistent with the distribution of high biomass and production were three regions of high assimilation numbers (Fig 2C), one off the mouth of the Xiaoling River, a second roughly 40 km to the southwest of the mouth of the Liaohe River, and a third about 30 km offshore and southwest of the mouth of the Daliao River.

Concentrations of DIN (Fig 2D) and silicate (Fig 2E) were above concentrations that would be limiting to phytoplankton growth throughout the bay [40]. Phosphate concentrations (Fig 2F) were high in the central part of the bay but were less than 25 nM at a total of 10 stations in the northwest and southeast sectors of the bay. Continuous culture studies of phytoplankton under phosphate-limited conditions have revealed half-saturation constants for growth that average 11 nM and in all cases are <30 nM [45–48]. Thus phytoplankton growth rates may have been phosphate limited to some extent in the northwest and southeast sectors of the bay, but in the remainder of Liaodong Bay phytoplankton growth appears to have been unconstrained by nutrient concentrations.

**Fig 3:**
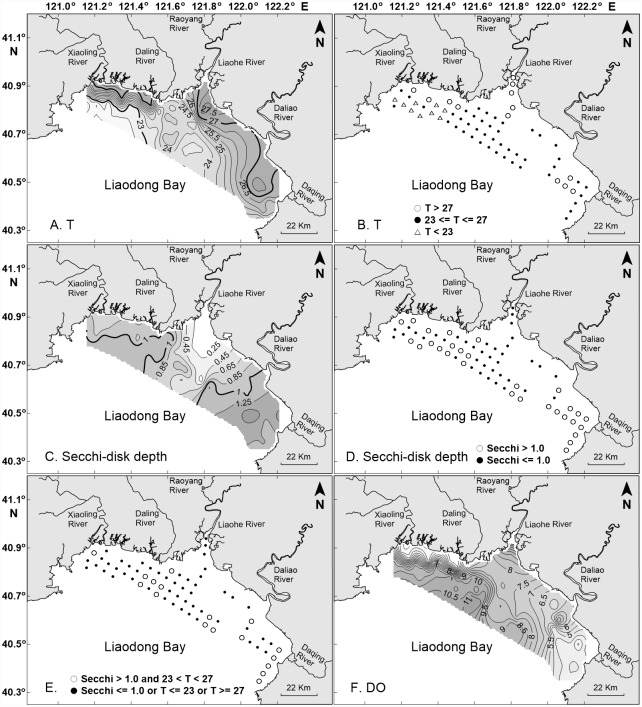
Contour maps and/or corresponding location maps of temperature (Panels A and B, in °C), Secchi-disk depth (Panels C and D, in meters), Secchi-disk depth associated with temperature (Panel E), and dissolved oxygen (DO, Panel F, in mg L^–1^).

Temperature and irradiance appear to have been the principal factors limiting photosynthetic rates throughout most of Liaodong Bay. Studies by Pei et al. [40] have shown that the assimilation numbers of phytoplankton in Liaodong Bay are functions of temperature and are highest in the temperature range 23–27°C. Surface temperatures in Liaodong Bay were either below or above this optimal range in three areas (Fig 3A and 3B): the estuary of the Liaohe River, most of the northwest sector of the bay, and a patch centered roughly 15 km off the mouth of the Daqing River in the southeast sector of the bay. The irradiance available for photosynthesis varied as a function of light transmission through the water column. Pei et al. [40] assumed the depth of the euphotic zone in the bay to be twice the Secchi depth. The mean and median Secchi depths in Liaodong Bay were 0.9 and 1.0 m, respectively. Secchi depths (Fig 3C and 3D) were greater than 1.0 m throughout the southeast sector of the bay, in a patch roughly 30 km offshore and equidistant from the mouths of the Liaohe and Daliao rivers, throughout much of the bay off the mouth of the Daling River, and in a small patch directly off the mouth of the Xiaoling River. Combining the information on temperature and Secchi depths revealed five patches where the surface temperature was in the range 23–27°C and Secchi depths were greater than 1 m (Fig 3E): a small patch near the mouth of the Xiaoling River, a much larger patch off the mouth of the Daling River, a somewhat smaller patch about 30 km offshore and equidistant from the mouths of the Liaohe and Daliao rivers, a patch about 15 km off the mouth of the Daliao River, and a linear region along the shoreline adjacent to the mouth of the Daqing River. These patches of optimal temperature and Secchi depths greater than 1 m corresponded closely to the patches of high areal photosynthetic rates, with the exception of the linear region along the shoreline adjacent to the mouth of the Daqing River.

The surface waters of the bay were undersaturated with respect to oxygen at 32 of the 66 sampling stations (Fig 3F). Patches of oxygen-undersaturated water were apparent off the mouths of each of the five major rivers that discharge to the bay. The largest patches of undersaturated water, 9–10 stations each, were present off the mouths of the Liaohe and Daqing rivers.

**Fig 4:**
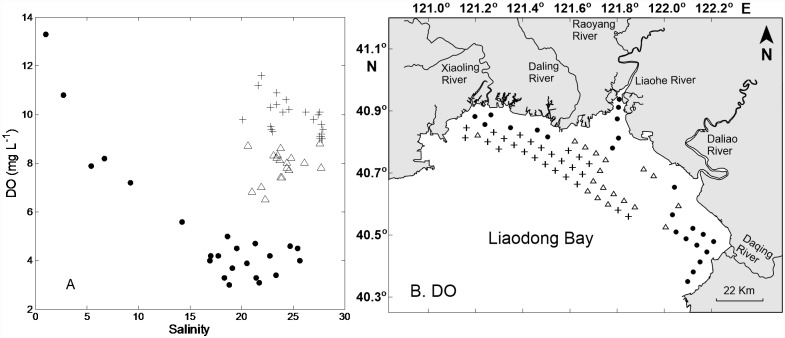
Relationship between DO concentrations and salinity (Panel A) and location of stations in three groups with different DO concentrations (Panel B). In Panels A and B, circle dots denote stations with high DO of more than 13 mg L^–1^ at a salinity of 1 to low DO concentrations of 3–5 mg L^–1^ at salinities of 16–26; Triangles and plus signs denote stations with DO concentrations of roughly 7–9 mg L^–1^ and 9–11 mg L^–1^, respectively, in both cases at salinities of 20–28.

Examination of the relationship between dissolved oxygen (DO) concentrations and salinity (Fig 4A) revealed three more-or-less distinct relationships. At one set of stations salinities and DO concentrations were negatively correlated from a high DO of more than 13 mg L^–1^ at a salinity of 1 to low DO concentrations of 3–5 mg L^–1^ at salinities of 16–26. The second and third sets of stations were associated with DO concentrations of roughly 7–9 mg L^–1^ and 9–11 mg L^–1^, respectively, in both cases at salinities of 20–28. Examination of the location of these stations revealed that the stations in the first group were all located in the estuary of the Liaohe River or along the coastline adjacent to the mouths of streams that discharge to Liaodong Bay (Fig 4B). The stations in the second group were located primarily offshore and east of 121.6°E longitude. The stations in the third group were all located offshore and west of 121.6°E longitude. Thus, in terms of the oxygen regime in the surface water, each station in Liaodong Bay could be assigned to one of three groups, one group being associated with stream mouths, and the other two being in most cases offshore and either east or west of 121.6°E longitude. The difference between the DO concentrations in the two groups of offshore stations was statistically significant (*t*-test, p<10^−9^) and was not explained by differences of oxygen solubility; the mean percentages of DO saturation in the offshore stations west and east of 121.6°E were 117% and 99%, respectively, the difference being significant at *p*<10^−6^. Mean Secchi depths at the offshore stations west and east of 121.6°E were 0.95 and 0.74 m, respectively, the difference being significant at *p* = 0.02. Thus the light regime in the water column was more favorable for phytoplankton growth west of 121.6°E. Not surprisingly, the mean areal photosynthetic rates in the western group of offshore stations, 1183 mg C m^–2^ d^–1^, was higher than the mean of the eastern group, 822 mg C m^–2^ d^–1^, but the difference was not statistically significant (Kruskal-Wallis test, *p* = 0.11).

### Surface waters versus bottom waters

A paired *t*-test revealed a significant (*p*<10^−6^) positive difference between the temperatures of surface and bottom waters. The greatest thermal stratification occurred in the southeastern part of the bay, where the difference in the temperature of surface and bottom waters consistently exceeded 1.3°C.

Salinities were likewise significantly different between surface and bottom samples (*t*-test, *p*<10^−4^). The differences were greatest in the southeastern part of the bay, where bottom salinities exceeded surface salinities by more than 1. At all but six stations in the remainder of the bay, bottom salinities exceeded surface salinities by less than 1, and surface salinities exceeded bottom salinities at 20 stations in the bay.

Consistent with the distribution of temperature and salinity, the difference in potential density anomaly, σ_θ_, between the surface and bottom water samples was greatest in the southern half of the bay, where the σ_θ_ of the bottom water exceeded the σ_θ_ of the surface water by more than 0.125 kg m^–3^. In the part of the bay north of 47°N, the difference between the σ_θ_ of bottom and surface water was less than 0.125 kg m^–3^ at roughly half the stations. Because the bottom of the mixed layer is customarily defined as the depth where the σ_θ_ of the water exceeds the σ_θ_ of the surface water by 0.125 kg m^–3^ [49], the mixed layer included the entire water column in roughly half of the northern part of the bay, whereas the bottom was below the mixed layer in more than 90% of the southern part of the bay.

Surface concentrations of nitrate and Chl *a* exceeded bottom water concentrations at 71% of the stations in the bay, the average differences being 5 μM and 1.0 mg m^–3^. DO concentrations and percent DO saturation were higher in surface water than in bottom water at 61% of the stations in the bay; bottom water concentrations and percent saturation were higher off the mouths of the Liaohe and Daqing rivers. The minimum DO concentration and percent saturation were approximately 3 mg L^–1^ and 42%, respectively, in both surface and bottom water. The stations associated with these low concentrations were directly adjacent to the mouth of the Daqing River. Ammonium and phosphate concentrations were not significantly different in surface and bottom waters (*t*-test, p = 0.69 and 0.50, respectively). SPM concentrations were 73.6 and 108 mg L^–1^ in the surface and bottom waters, respectively, and they were higher in bottom water than surface water (*t*-test, *p* = 0.0012) at 67% of the stations in the bay, particularly off the mouth of the Liaohe River.

## Discussion

Despite the shallowness of Liaodong Bay and the fact that the tidal amplitude ranges between 0.8 and 2.7 m, the bottom waters of substantial parts of the bay were below the depth of the mixed layer as it is traditionally defined in terms of differences in potential density [49]. In particular, the bottom waters of more than 90% of the stations at latitudes less than 40.7°N were below the depth of the mixed layer. In contrast, the entire water columns of about 57% the stations at latitudes greater than 40.7°N were within the mixed layer. Thus, during the time of our study, Liaodong Bay could be logically divided into two sectors, a southern sector below 40.7°N, within which almost all bottom waters were below the depth of the mixed layer, and a northern sector above 40.7°N within which about half the stations were well mixed. This division of the bay was directly related to the average depths of the water column in the two sectors, which were 4.2 m and 5.6 m in the northern and southern sectors, respectively.

Because light and temperature appear to be the principal factors constraining photosynthetic rates in the bay, the patterns of primary production were relatively easy to explain on the basis of the distribution of temperature and Secchi depths. Temperatures were within the optimal range of 23–27°C at all stations in the central part of the bay and adjacent to the southeastern shoreline. Temperatures were supraoptimal (>27°C) in the estuary of the Liaohe River, near the mouths of the Xiaoling and Daling rivers, and at four stations intermediate between the mouths of the Daliao and Daqing rivers. Temperatures were suboptimal (<23°C) at most of the offshore stations in the northwestern part of the bay.

Superimposed on this pattern was the distribution of Secchi depths, which were less than 1 m at many of the stations that were optimal in terms of temperature, including all stations in the central part of the bay directly off the mouth of the Liaohe River, a reflection of the high concentrations of SPM in the Liaohe River. The result was that there were three patches of water in the bay that were optimal for photosynthesis in terms of temperature and light. Two of those patches were offshore to the northwest and southeast of the plume of turbid water from the Liaohe River. The third patch was located directly near the shoreline in the southeastern part of the bay adjacent to the mouth of the Daqing River. Examination of the distribution of photosynthetic rates (Fig 2A) and Chl *a* (Fig 2B) in the bay revealed offshore patches of high production and biomass in the approximate locations predicted by the distribution of temperature and Secchi depths. However, there was no evidence of high photosynthetic rates along the shoreline adjacent to the mouth of the Daqing River. The most likely explanation for the failure of a patch of high production to appear in this area is the fact that the size of the patch of favorable growth conditions in the onshore-offshore direction was too small to sustain a bloom of phytoplankton. Even at a phytoplankton growth rate of 1 d^–1^, the critical patch size required to overcome losses to turbulent diffusion is roughly 1–2 km [9], and herbivore grazing would increase the critical patch size further [50]. The dimensions of the patches of favorable growth conditions in the offshore waters of the bay were roughly 10 km and therefore more than adequate to support development of phytoplankton blooms. Pollution from the Daqing River water may be a contributing factor. The downstream waters of the Daqing River runs through cities [51], and sanitary and industrial sewage with large amounts of petroleum pollutants and anionic surfactants have adversely impacted water quality in the river. However, there is no information on the effects of these contaminants on the coastal phytoplankton community in Liaodong Bay to date.

Previous studies have suggested that diatoms such as *Nitzschia spp*., *Nitzschia longissima* (Breb.) Ralfs, and *Coscinodiscus oculus-iridis* Ehrenberg are the dominant species in summer in Liaodong Bay [52, 53]. However, there has been a shift of the phytoplankton community from diatom domination to coexistence of diatoms and dinoflagellates in the past decades in Liaodong Bay [54] and in the central Bohai Sea [29, 55]. Correspondingly, the frequency of occurrence of HABs has increased drastically in the Bohai Sea (including Liaodong Bay), and the Bohai Sea has become one of the main areas of frequent HAB occurrences along the coast of China [56]. An especially serious HAB took place during late September and early October of 1998 in the western and central sectors of Liaodong Bay; the bloom covered over 5000 km^2^ and caused severe economic losses [57]. Much discussion has concerned the causes of the increasing frequency of HABs.

Some studies [24, 26] have shown that the Liaodong Bay ecosystem has been stressed by eutrophication, and conditions have become more serious in the coastline around the estuaries of the Daliao, Liaohe, and Daling rivers as a result of excessive nutrient inputs. It has also been hypothesized that the increasing N/P ratio and decreasing Si/N ratio in the Bohai Sea since the 1980s caused by human activities might be responsible for the shift of the phytoplankton community toward dinoflagellates [29, 55]. In our study, nutrient concentrations exceeded growth-rate-saturating concentrations throughout the bay, with the possible exception of phosphate at some stations, implying that other factors (principally, temperature and irradiance) rather than nutrients are becoming the principal factors controlling photosynthetic rates throughout most of Liaodong Bay. Since dinoflagellates are motile and can move to the surface, they have an advantage over diatoms in a seriously light-limited system [58]. Taken together, light-limitation might be becoming one of the major drivers for the shift of the phytoplankton community in Liaodong Bay [54]. Comparatively, such a shift in central Bohai Sea might be caused primarily by the change of nutrient ratios [29, 55], zooplankton grazing pressure [59] and the effects of heavy metal and oil pollution.

Observations of phytoplankton patches and examination of their mechanisms of formation might provide clues as to why HABs have become more frequent. However, phytoplankton patchiness and thin layers have been studied less in the coastal areas of China compared, for example, with the Layered Organization in the Coastal Ocean project carried out in Monterey Bay, California, a study that has involved intensive multi-investigator efforts [32, 60]. A similar recent investigation was conducted during the spring inter-monsoon season along the western coast of the South China Sea [61]; that study revealed that formation of phytoplankton patches as well as nutrient and temperature variations were related to wind-stress curl, and the spatial distribution and vertical structure of patchiness were governed by nutrients advected by curl-driven upwelling through Ekman pumping [61]. Patches of phytoplankton thin layers have been observed annually in Monterey Bay, but large differences in the dominant algal species, persistence of the patches, vertical position in the water column, and integrated chlorophyll concentrations resulted from differences of biological and physical dynamics during the years 2002, 2005, and 2006 [62].

In Liaodong Bay, phytoplankton patchiness may also be affected by diel phenomena, as suggested by the observations of Benoit-Bird et al. [63], the shift of taxonomic composition from non-motile diatoms to motile dinoflagellates similar to the observations in Monterey Bay [64], and physical oceanographic processes, such as turbulent mixing and shear stress. Li et al. [65] found there was obvious diurnal rhythm of phytoplankton community composition in the surf zone of Daliao River estuary in the spring of 2013, and the percentage of diatom in phytoplankton community reached the minimum at 0:00 and 12:00, comparatively, the peak of biomass appeared at 16:00. In addition, both human activities and changing climate are causing multiple stressors to the coastal and oceanic ecosystem and modifying their productivities [66]. Although Liaodong Bay is one of three bays in the Bohai Sea and relatively small compared with the long Chinese coastline, it is very productive in terms of fisheries and of significant economic importance to northeastern China. The bay provides an ideal opportunity for further studies of ecological issues impacted by the combined effects of human activities and environmental change.

## Conclusions

In this study, several patches of high biomass and production with horizontal dimensions of roughly 10 km were apparent from examination of areal photosynthetic rates and concentrations of Chl *a*. Three regions of high assimilation numbers were observed with locations consistent with the areas of high biomass and production. Phytoplankton growth rates seem not to be limited by nutrient concentrations, with the exception of a few stations in the northwest and southeast sectors of the bay. Instead, phytoplankton growth rates are controlled by the distribution of water temperature and variation of light penetration restricted by water turbidity. Combining information on temperature and Secchi depths revealed five patches where surface temperatures were in the range 23–27°C and Secchi depths were greater than 1 m. These patches of optimal conditions for phytoplankton growth corresponded closely to the patches of high areal photosynthetic rates, with the exception of a linear region along the shoreline adjacent to the mouth of the Daqing River. Based on the relationship between DO concentrations and salinity as well as the spatial distribution of oxygen in the surface water, the sampling stations of Liaodong Bay could be divided into three groups. The stations in the third group, located offshore and west of 121.6°E longitude, were the most favorable for phytoplankton growth as a result of their greater Secchi depths and hence better light regime. Bottom waters of nearly all of the eastern bay were below the depth of the mixed layer and were characterized by the lowest oxygen concentrations, 3–5 mg L^–1^. In contrast, the water column in much of the remainder of the bay was within the mixed layer, and oxygen concentrations were higher than 5 mg L^–1^ in both surface and bottom waters. Apparently, patches of phytoplankton in Liaodong Bay were determined to a large extent by temperature and light and not by nutrients in this study. As humans discharge more and more nutrients into coastal waters as a result of fertilizer usage and wastewater disposal, the importance of nutrients in the formation of patches and HABs will lessen. In contrast, the importance of light and temperature will increase under the growing influences from both human activities and climate change. Overall, our results complement earlier work by identifying factors that control the development and size of patches other than nutrients, and prove our hypotheses are correct, but with some caveats: the change from nutrient limitation to light and temperature limitation, combined with other factors of Si/N ratio shift, diel effects and the effect of toxic pollutants, may have favored dinoflagellates and HABs. Thus, our results provide a reference for coastal studies related to phytoplankton shift from diatoms to dinoflagellates, and the increasing problems of eutrophication, HABs and hypoxia.

## Supporting information

S1 Dataset (XLS)
